# Supplementary figures and images for: Plasma membrane recycling drives reservoir formation during Toxoplasma gondii intracellular replication
Source: PLoS Biol. 2025 Sep 30;23(9):e3003415. doi: 10.1371/journal.pbio.3003415 (PMC12503318; doi:10.1371/journal.pbio.3003415)

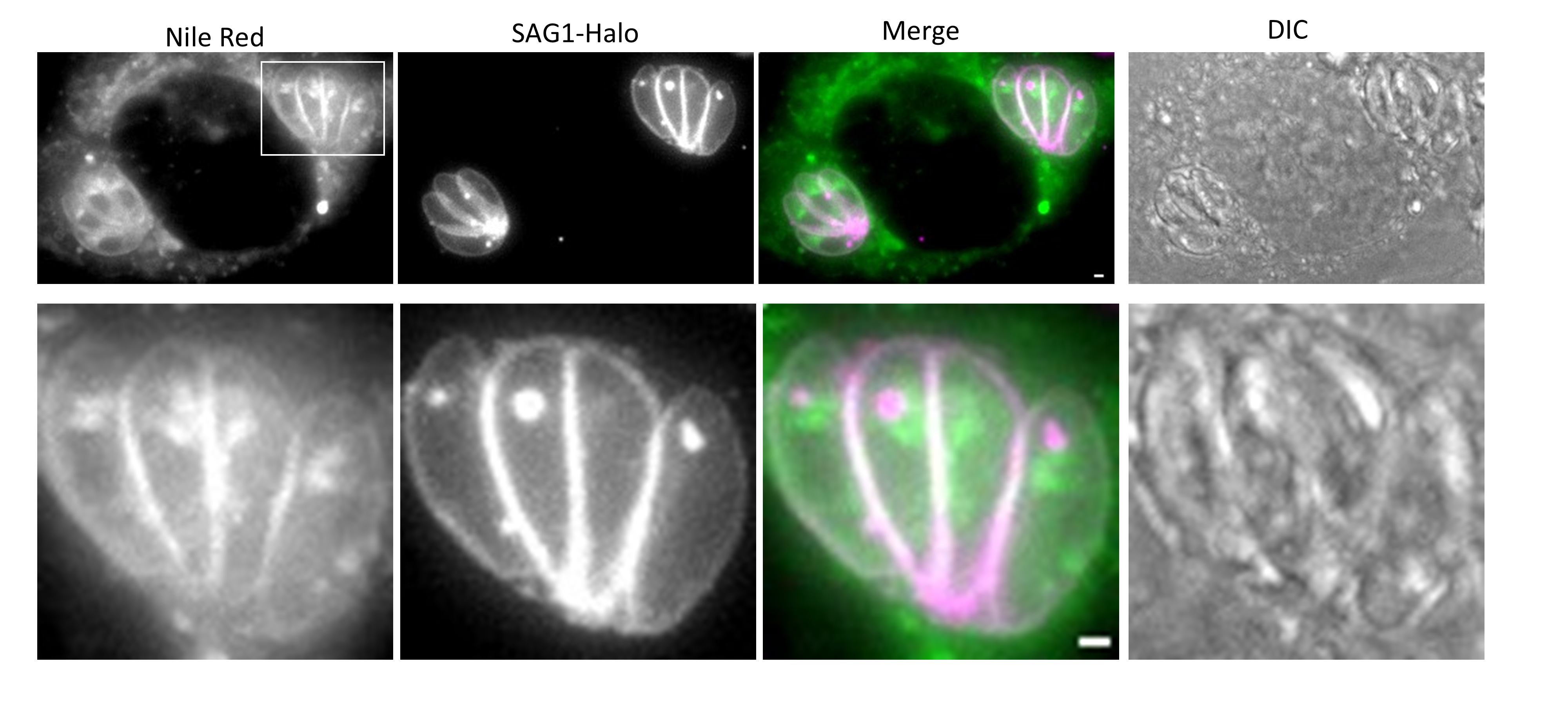

Supplement: S1 Fig — Representative picture of labeling with Nile Red and SAG1-Halo with Alexa 488. Scale bar 1 μm. (TIF) [file pbio.3003415.s001.TIF]

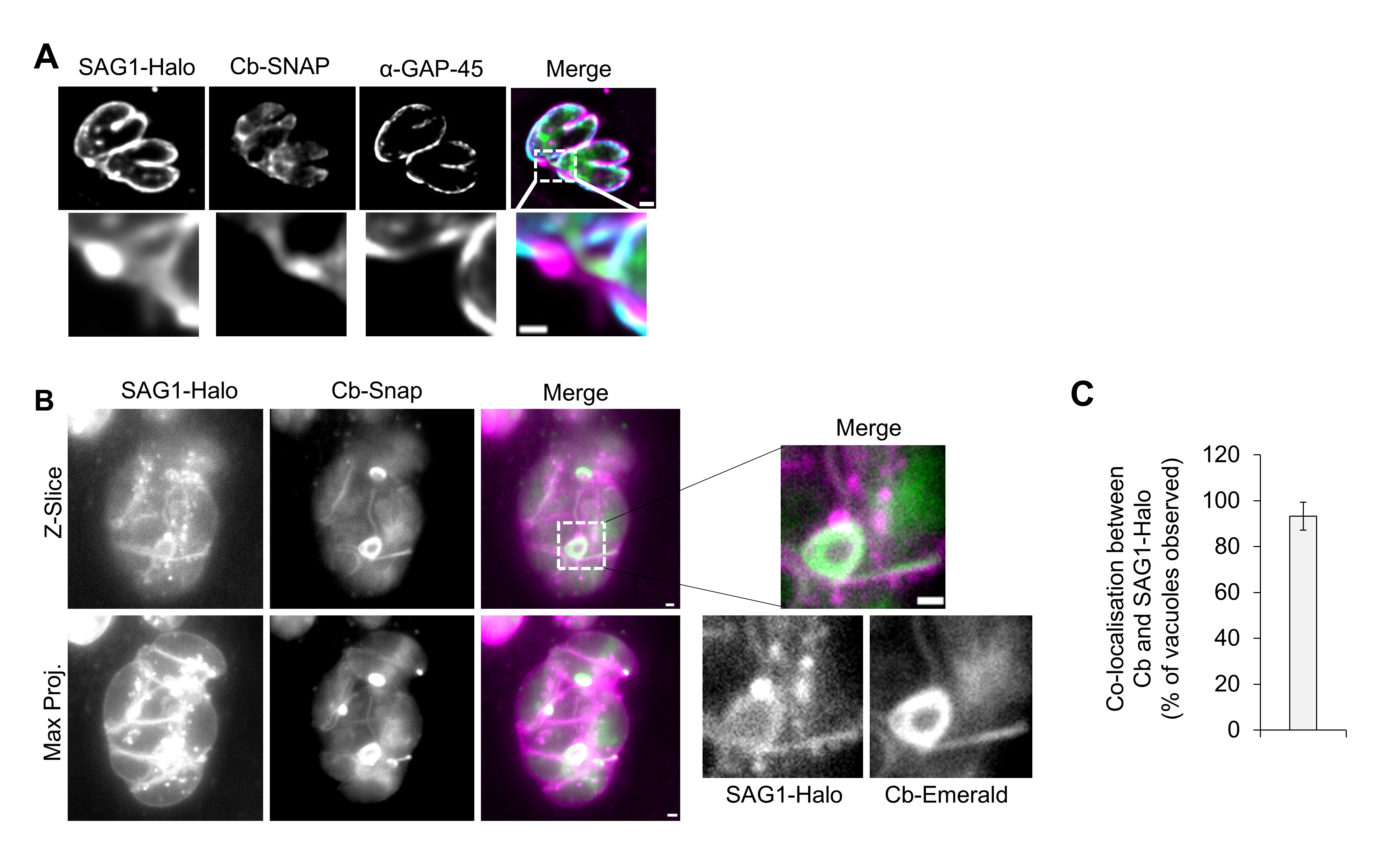

Supplement: S2 Fig — (A) The plasma membrane connection allowed the passage of F-actin from one parasite to the other. SAG1-Halo was visualized using a membrane-permeable dye Jan-647, the CB-snap with a membrane-permeable dye SNAP-594 and GAP45 using antibodies. The F-actin is located where SAG1-Halo is present, without any GAP45 staining. Scale bars 1 μm. (B) PM association with the F-actin network on larger vacuoles. SAG1-Halo was visualized using a membrane-permeable dye Jan-647 and the F-actin network with the expression of Cb-SNAP. Scale bars 1 μm. (C) Quantification of colocalisation between SAG1 Halo and Cb-SNAP. The percentage of vacuole where colocalisation between SAG1-Halo and Cb-SNAP was calculated. For each replicate, 25 vacuoles with visible F-actin network were selected and then analyzed for the presence of SAG1-Halo for the filament. Scale bars 1 μm. Three biological replicates were used for all analyses; Error bars are standard deviations and the center measurement of the graph bars is the mean. The data underlying this figure can be found in S1 Data. (TIF) [file pbio.3003415.s002.TIF]

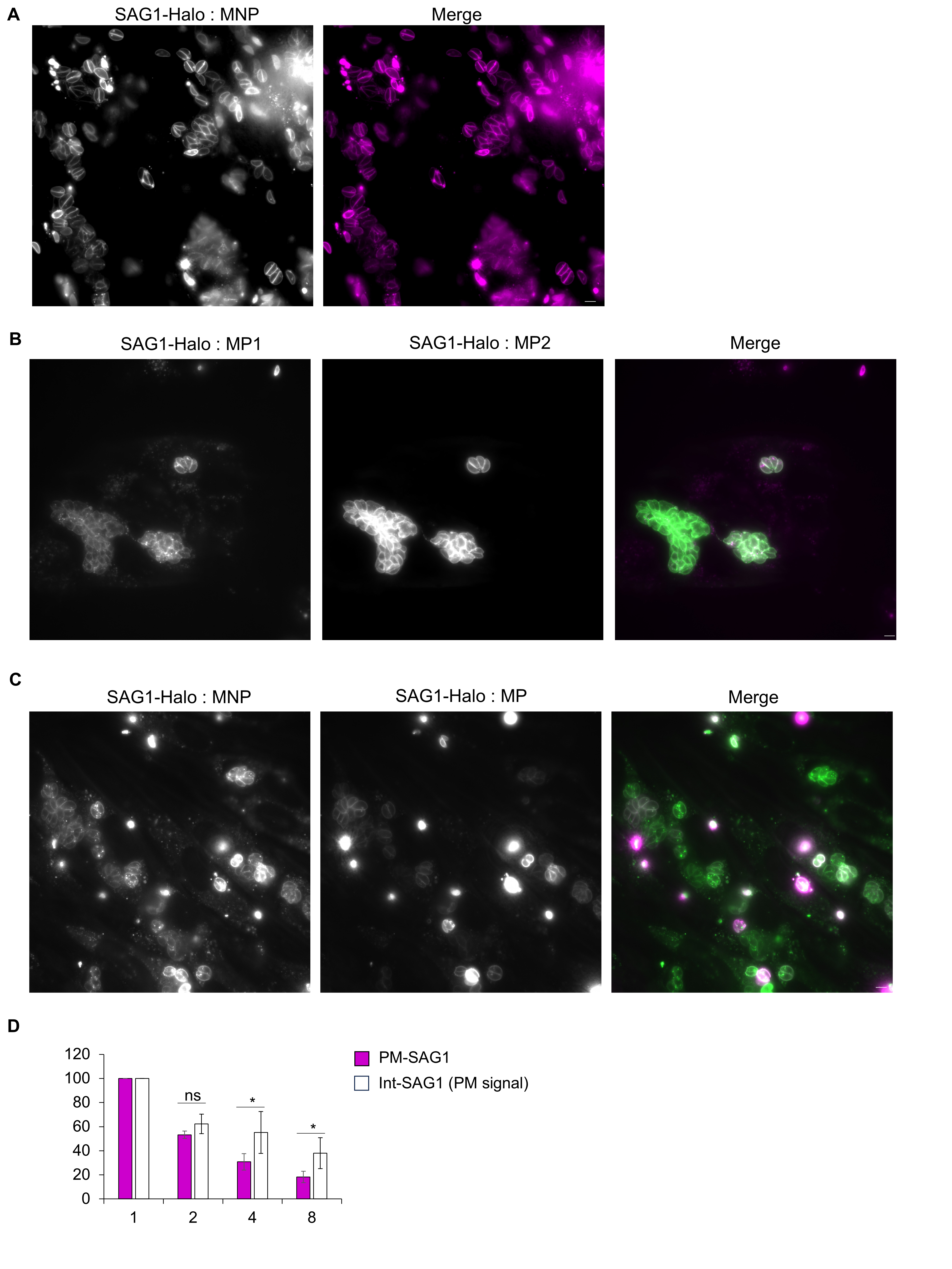

Supplement: S3 Fig — (A) Field of view (FOV) obtained with a membrane nonpermeable dye before the invasion, as illustrated in Fig 2A Top. Image taken after 24 h replication. (B) FOV obtained with a membrane-permeable dye before invasion and a second membrane-permeable dye after replication, as illustrated in Fig 2A middle. Image taken after 24 h replication. (C) FOV obtained with a membrane nonpermeable dye followed by a membrane-permeable dye before invasion, as illustrated in Fig 2A bottom. Image taken after 24 h replication. All scale bars = 5 µm. (D) Comparison of the plasma membrane signal between PM-SAG1 and Int-SAG1 during replication. Int-SAG1 decrease is slower than PM-SAG1. Three biological replicates were used for all analyses; all P values are 0 ≤ P ≤ 0.001, ***, error bars are standard deviations, and the center measurement of the graph bars is the mean. A one-tailed unpaired Student t test was used for all comparisons with no adjustments. The data underlying this figure can be found in S1 Data. (TIF) [file pbio.3003415.s003.TIF]

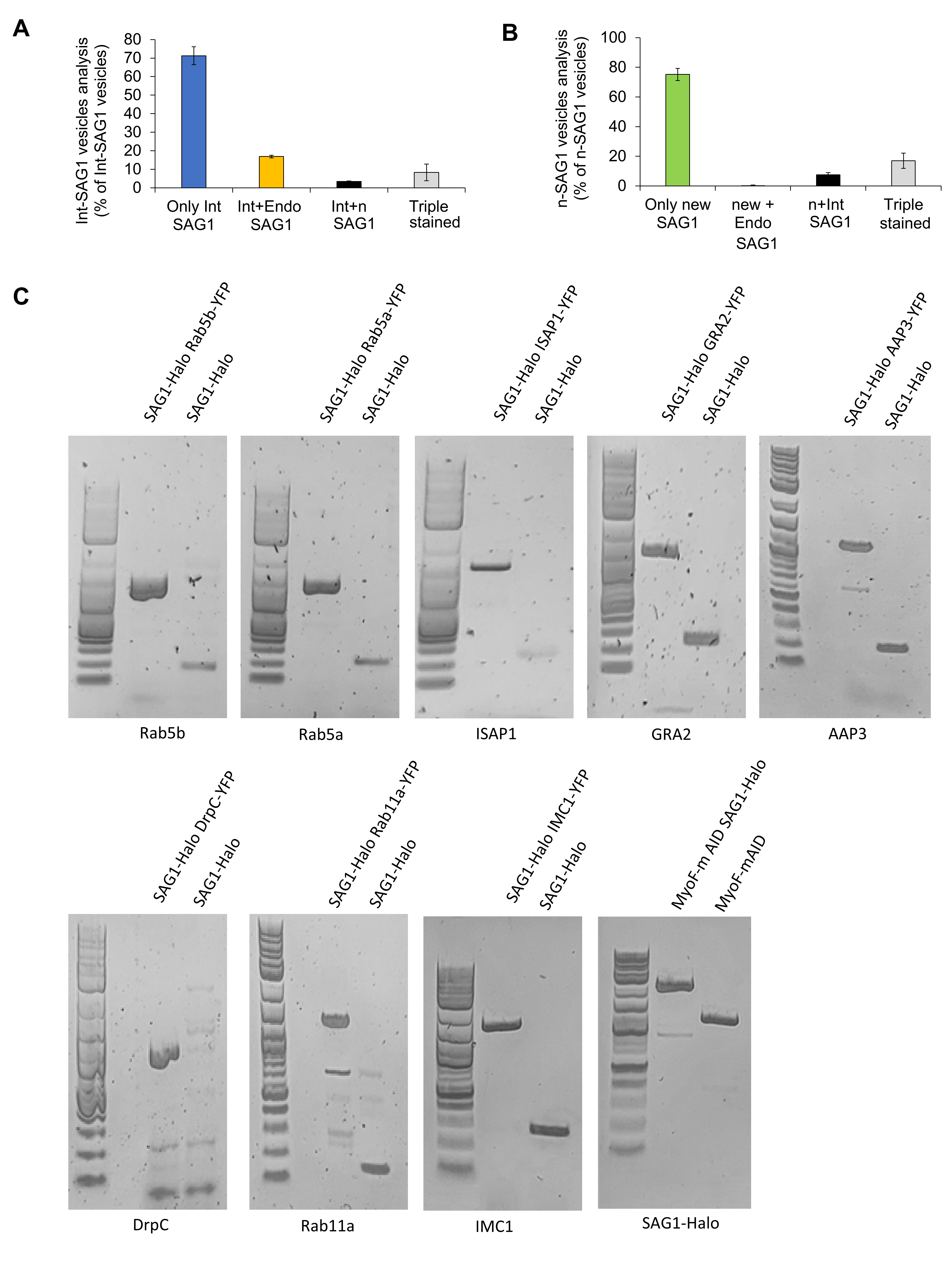

Supplement: S4 Fig — (A, B) Quantification of the colocalization between the different populations of SAG1 vesicles as performed in Fig 3C but taking Int-Sag1 (A) and n-SAG1 (B) as reference. (C) Integration PCR of the endogenous tagging performed for Figs 3D, 4, and 5. Three biological replicates were used for all analyses; all P values are 0 ≤ P ≤ 0.001, ***, error bars are standard deviations, and the center measurement of the graph bars is the mean. A one-tailed unpaired Student t test was used for all comparisons with no adjustments. The data underlying this figure can be found in S1 Data. (TIF) [file pbio.3003415.s004.TIF]

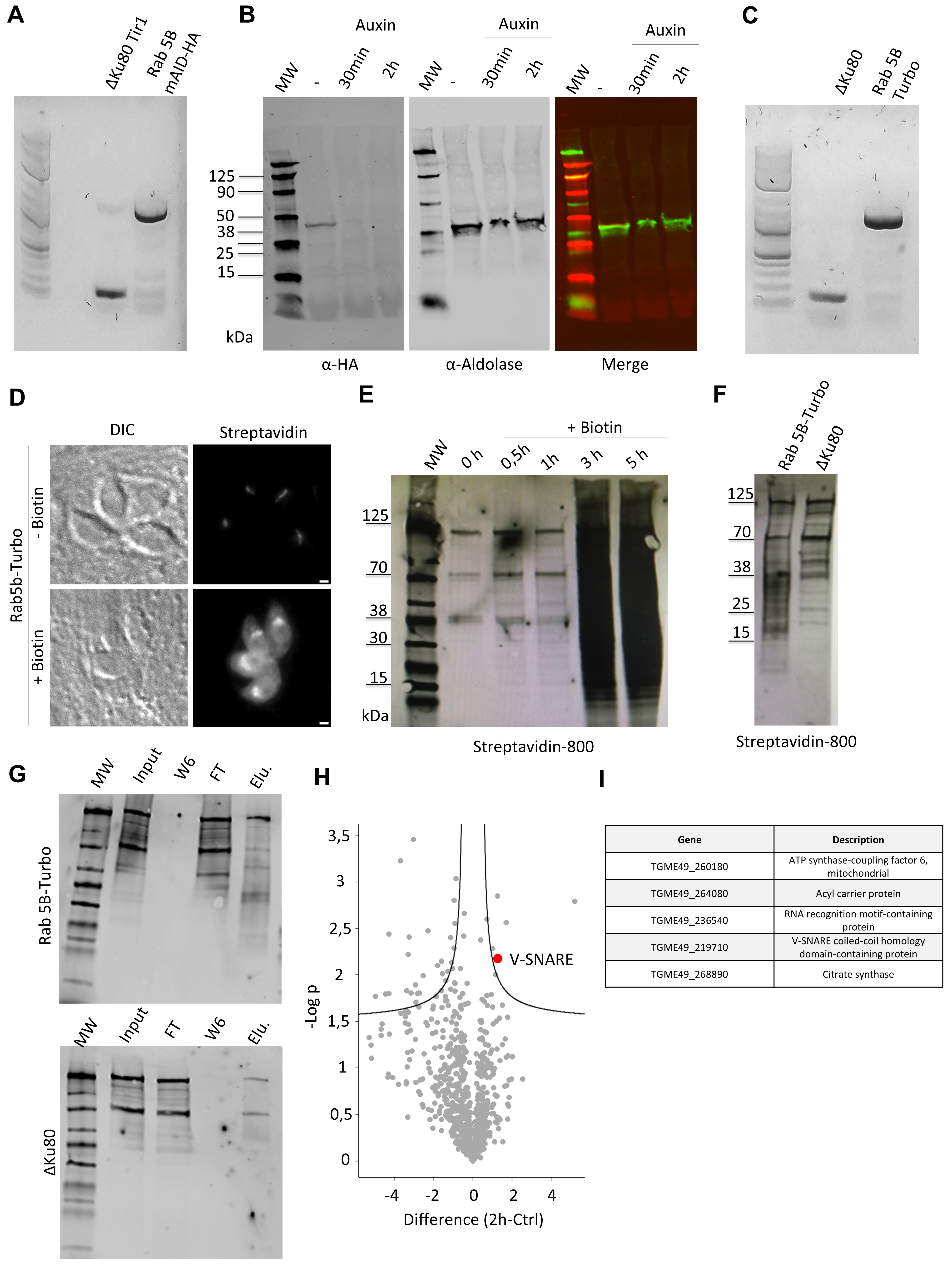

Supplement: S5 Fig — (A) Integration PCR of the mAID tag at the Rab5b locus. (B) Full western blot corresponding to the cropped version shown in Fig 4A. (C) Integration PCR of the TurboID tag at the Rab5b locus. (D) Representative images of Rab5b-TurboID activity ± biotin. (E) Full western blot showing the biotinylation profile of Rab5b-TurboID after various durations of biotin addition. (F) Comparison of biotinylation between Rab5b-TurboID and ΔKu80 after 2 h of biotin treatment. (G) Streptavidin pull-down comparison between Rab5b-TurboID and ΔKu80. (H) Volcano plot generated from mass spectrometry data. (I) List of biotinylated proteins enriched specifically in Rab5b-TurboID samples. All experiments were performed in three independent biological replicates. The data underlying this figure can be found in S1 Data. (TIF) [file pbio.3003415.s005.TIF]

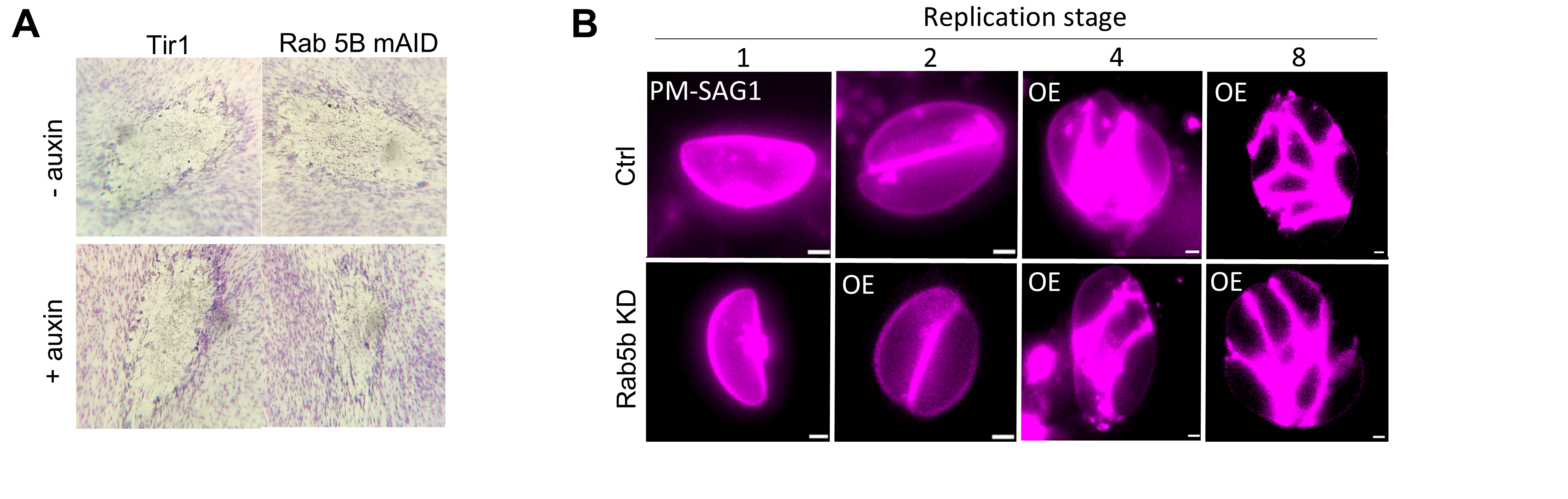

Supplement: S6 Fig — (A) Representative pictures of the plaque used for the plaque assay analysis in Fig 4B. (B) Overexposed version of the pictures used Fig 5G. The PM is still visible in all condition. (TIF) [file pbio.3003415.s006.TIF]

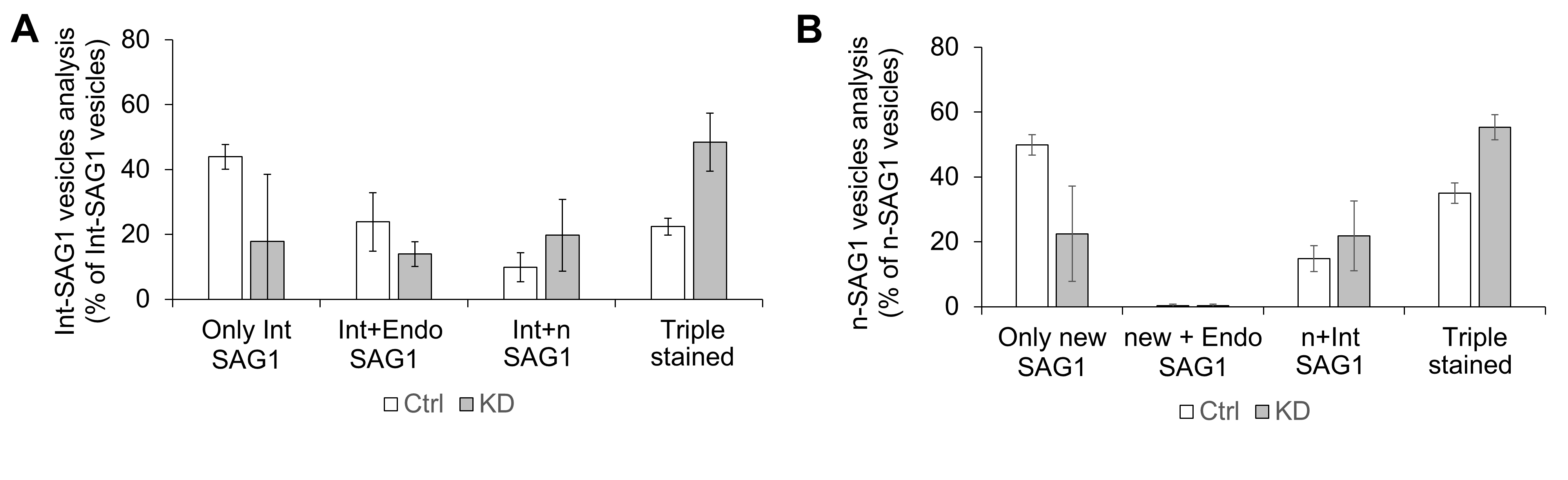

Supplement: S7 Fig — Quantification of the colocalization between the different populations of SAG1 vesicles as performed in Fig 4J, but taking Int-Sag1 (A) and n-SAG1 (B) as reference. White: MyoF ctrl/ − auxin, gray: MyoF KD/ + auxin. Three biological replicates were used for all analyses; all P values are 0 ≤ P ≤ 0.001, ***, error bars are standard deviations, and the center measurement of the graph bars is the mean. A one-tailed unpaired Student t test was used for all comparisons with no adjustments. The data underlying this figure can be found in S1 Data. (TIF) [file pbio.3003415.s007.TIF]

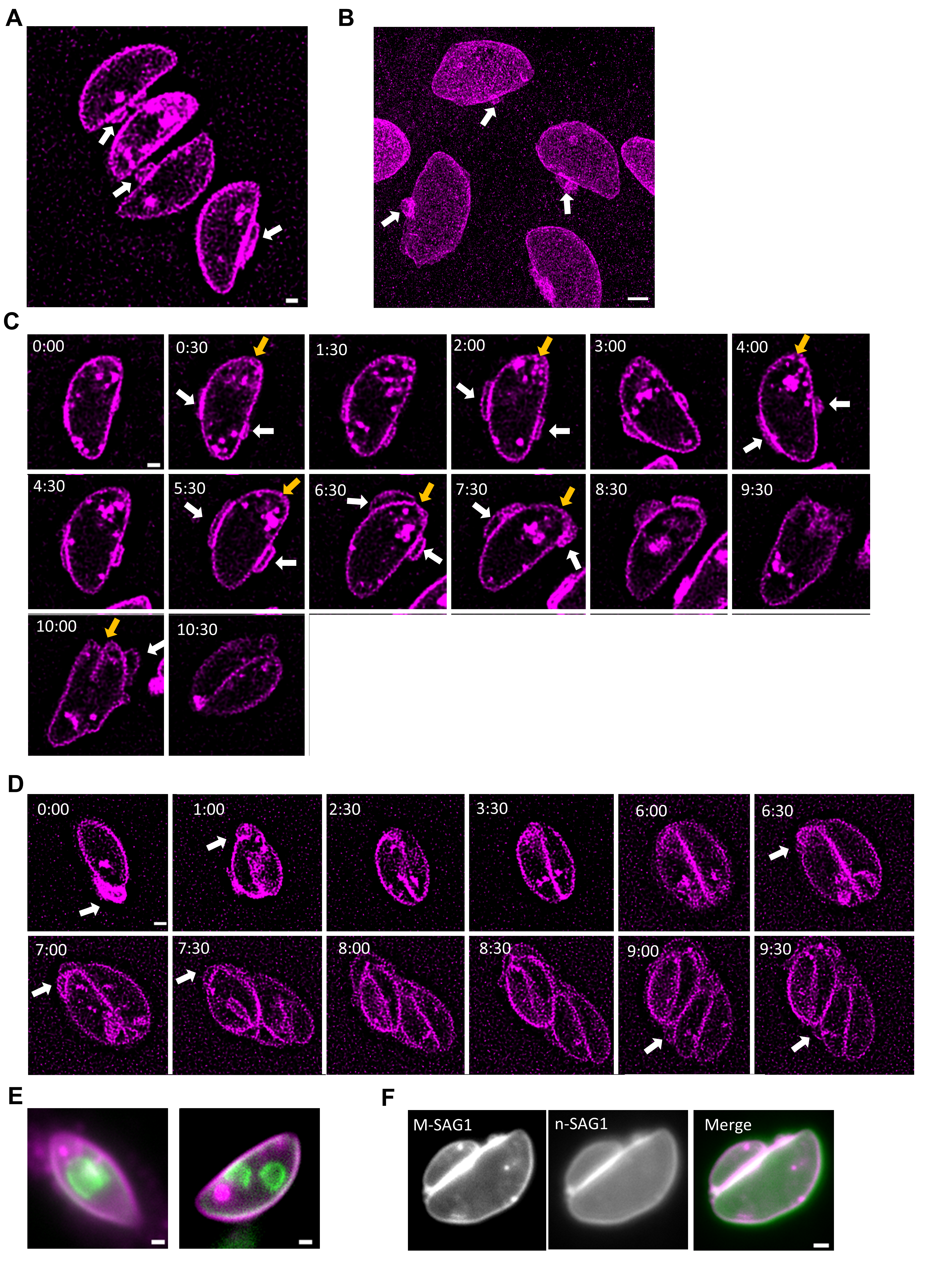

Supplement: S8 Fig — (A, B) The position of the plasma membrane reservoir (PMR) is not fixed. Fixed imaging of different parasites (A) SAG1-Halo or (B) RH with α-SAG1 in Ultra expansion microscopy. Multiple parasites present a PMR (white arrow) at different positions on the membrane. Scale bar 5 μm for Ultraexprension. (C) Live microscopy of the PMR dynamics. SAG1-Halo parasites were labeled and transferred to host cells for replication and live imaging. The top of the parasite is highlighted with a yellow arrow. The position of the PMRs is indicated by the white arrows. The position and the size of the PMRs are variable during the replication process. (D) Live replication of SAG1-Halo tachyzoite from stage 1 to 4. The PMR is highlighted with white arrows. (E) Tachyzoites with daughter cells form without visible PMR. Magenta: SAG1-Halo, Green: IMC1-YFP. (F) De novo material is present in the PMR. With the dual labeling with membrane-permeable dye to differentiate between maternal (M-SAG1) and de novo (n-SAG1) SAG1, n-SAG1 was detected to also form the PMR. All scale bars unless stated otherwise = 1 µm. (TIF) [file pbio.3003415.s008.TIF]
