# Supplementary material for: Plasma membrane recycling drives reservoir formation during Toxoplasma gondii intracellular replication
Source: PLoS Biol. 2025 Sep 30;23(9):e3003415. doi: 10.1371/journal.pbio.3003415 (PMC12503318; doi:10.1371/journal.pbio.3003415)

WB Figure 4A and S5B

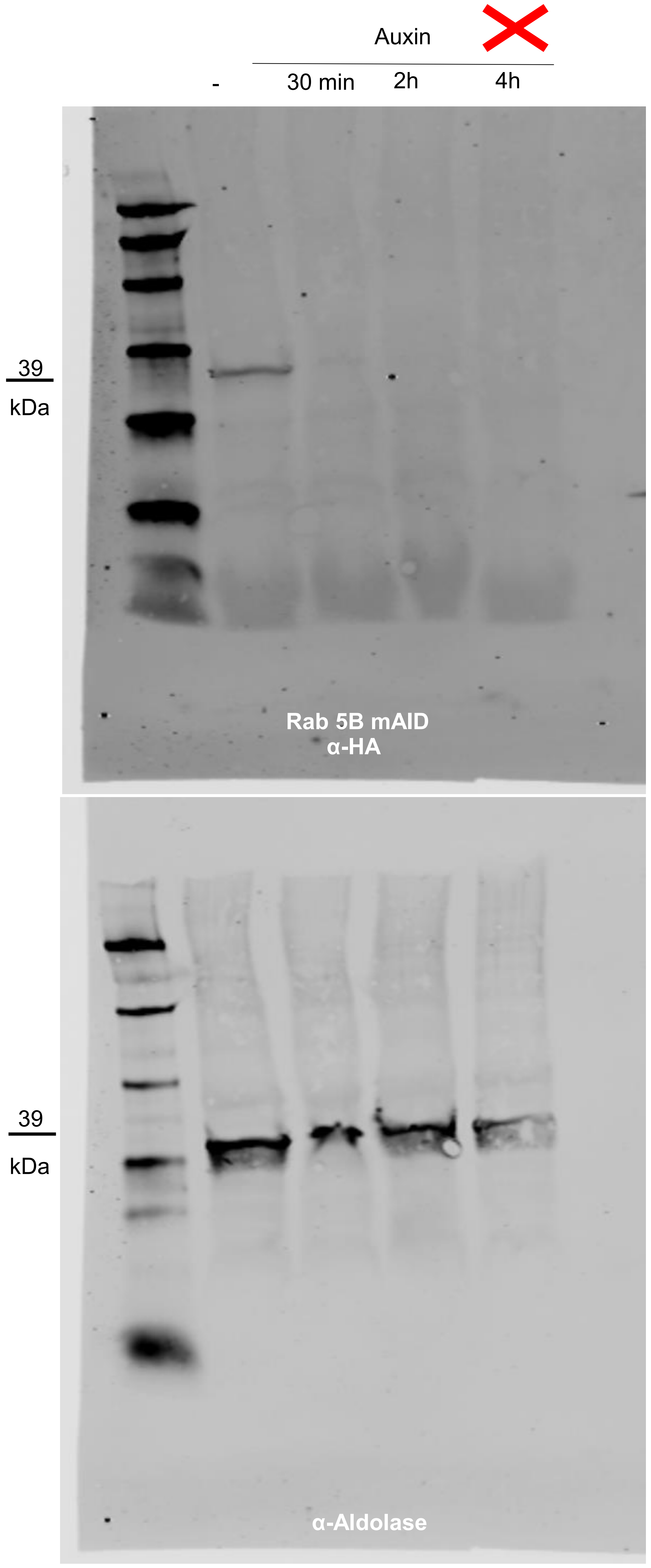

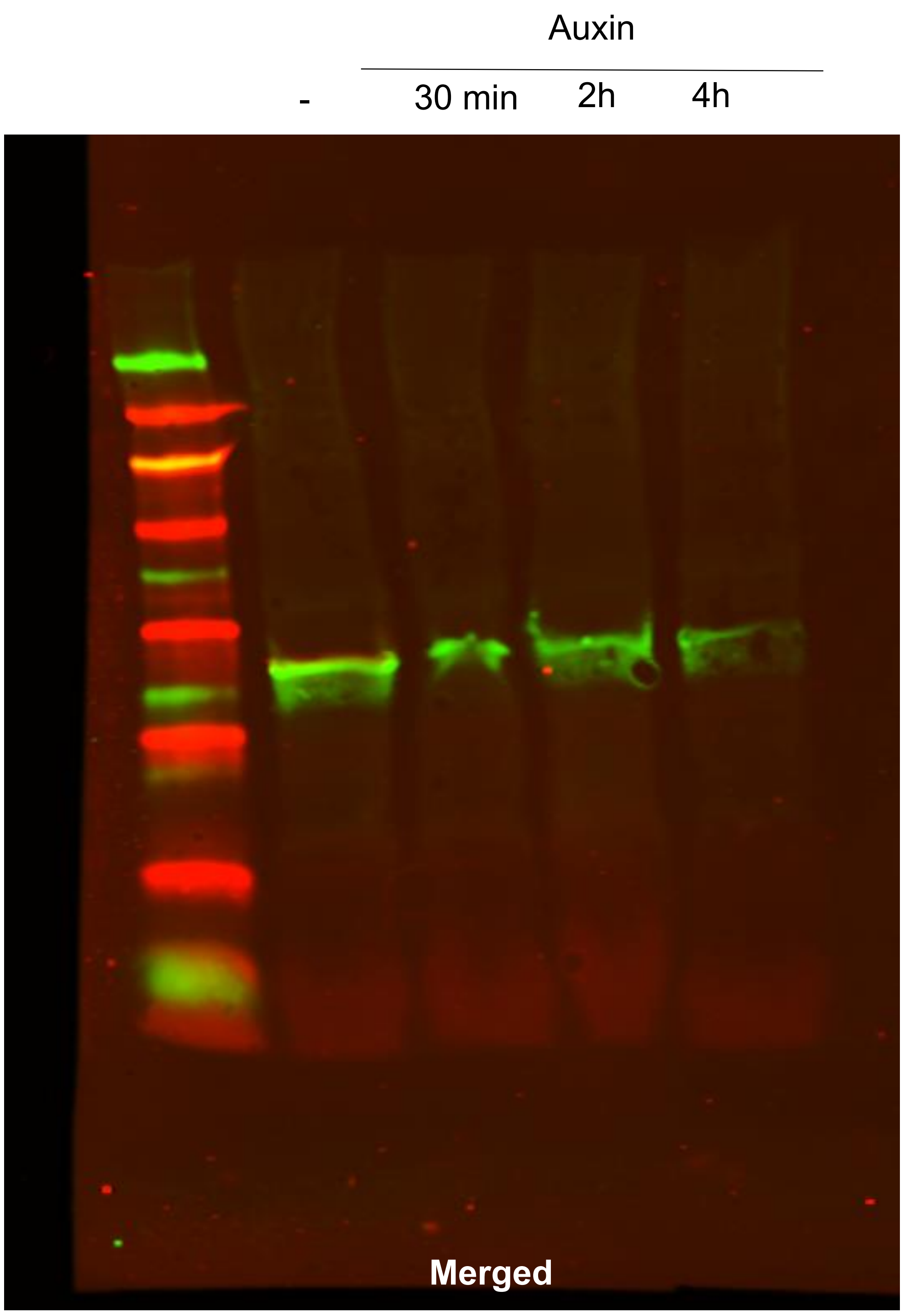

Not used in the paper figure

Integration PCR Gel Figure S4

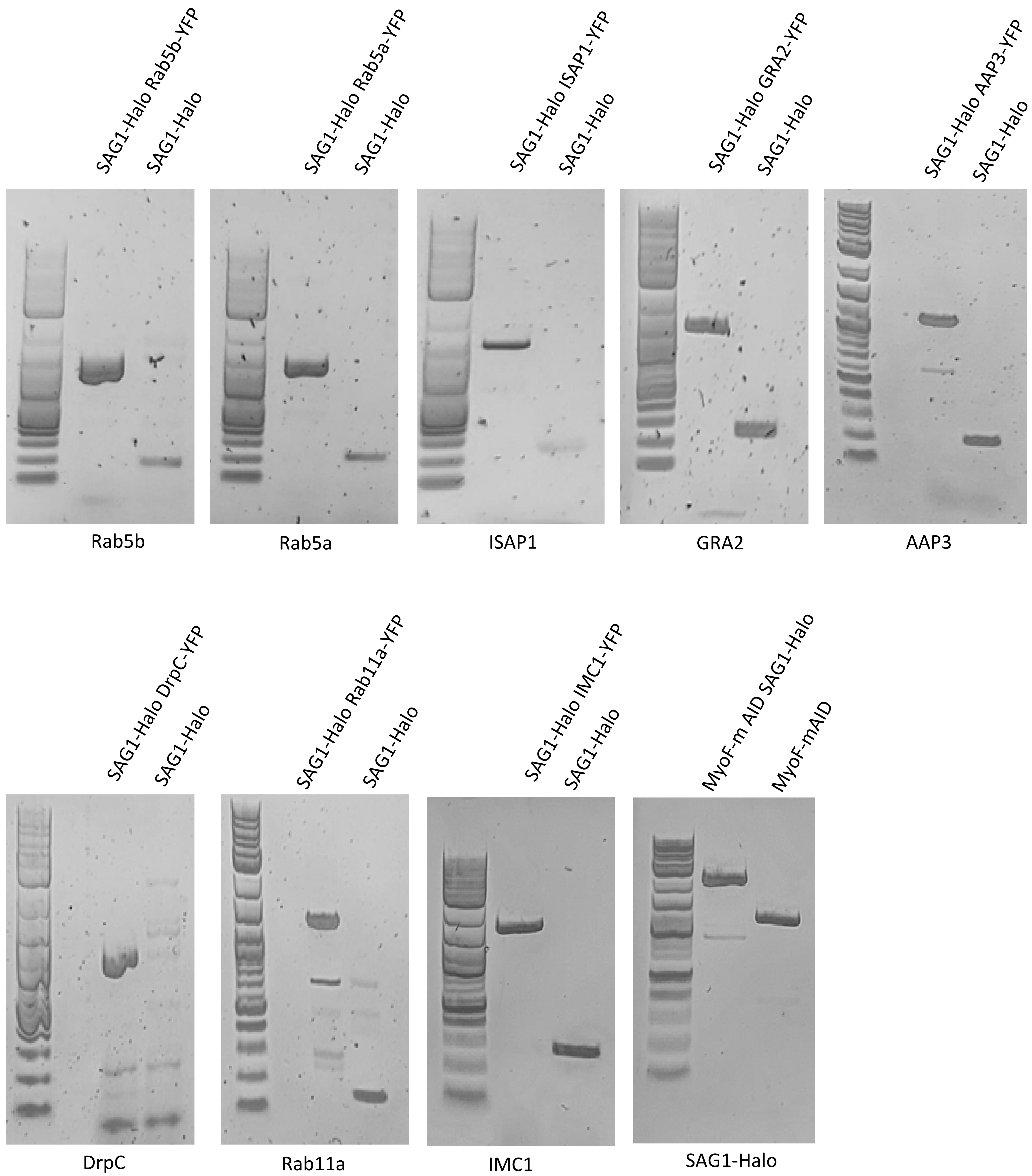

Integration PCR Gel Figure S5

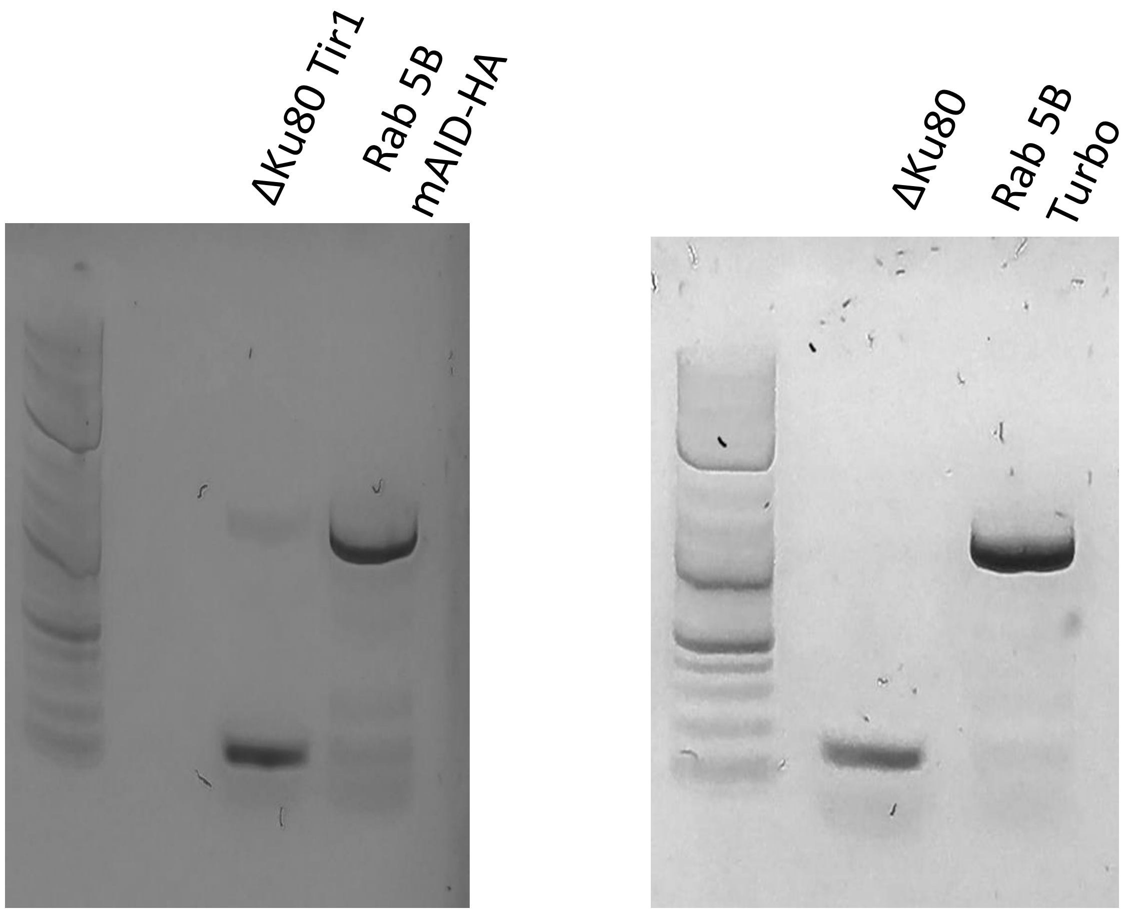

WB Figure S5E

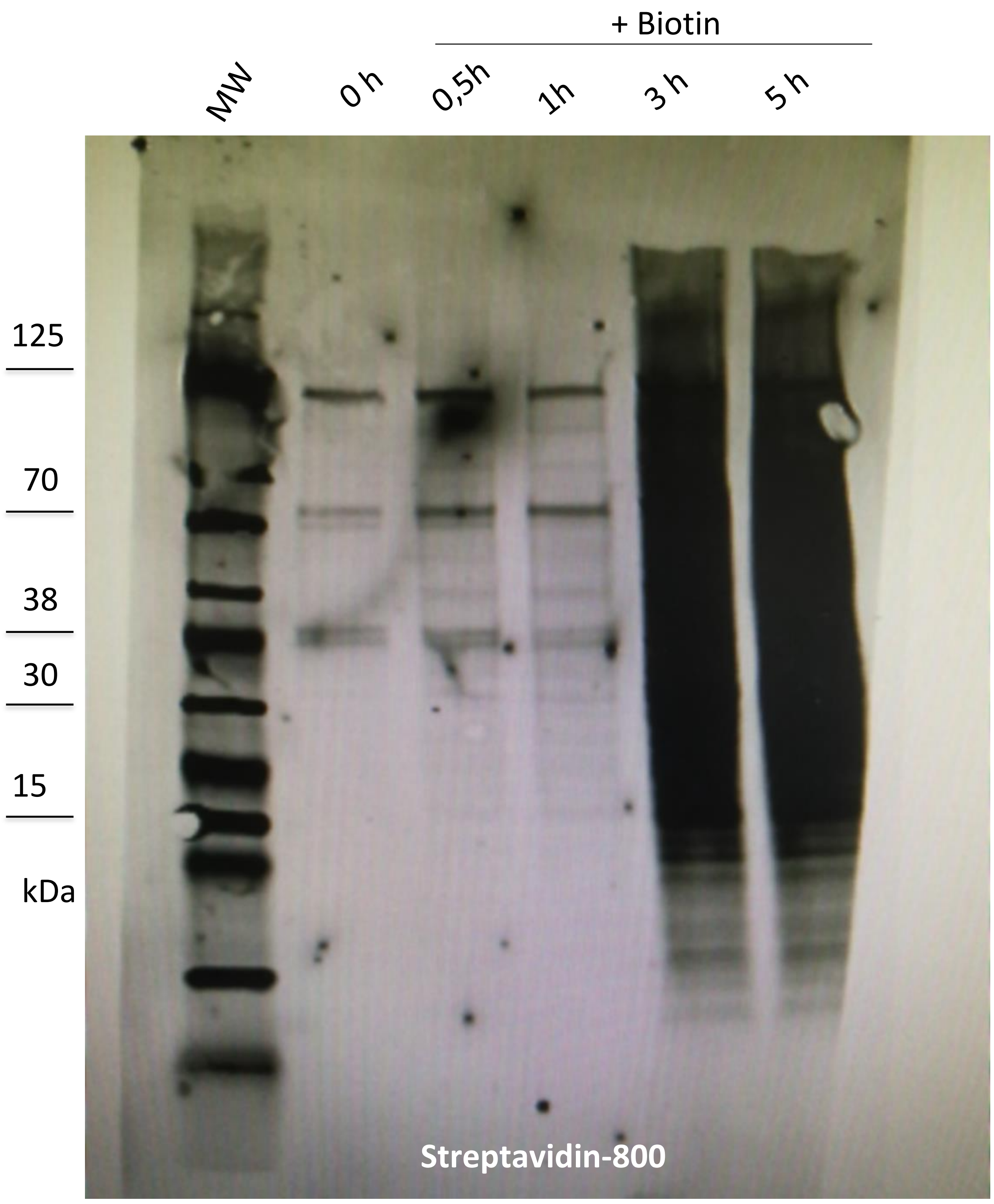

WB Figure S5F

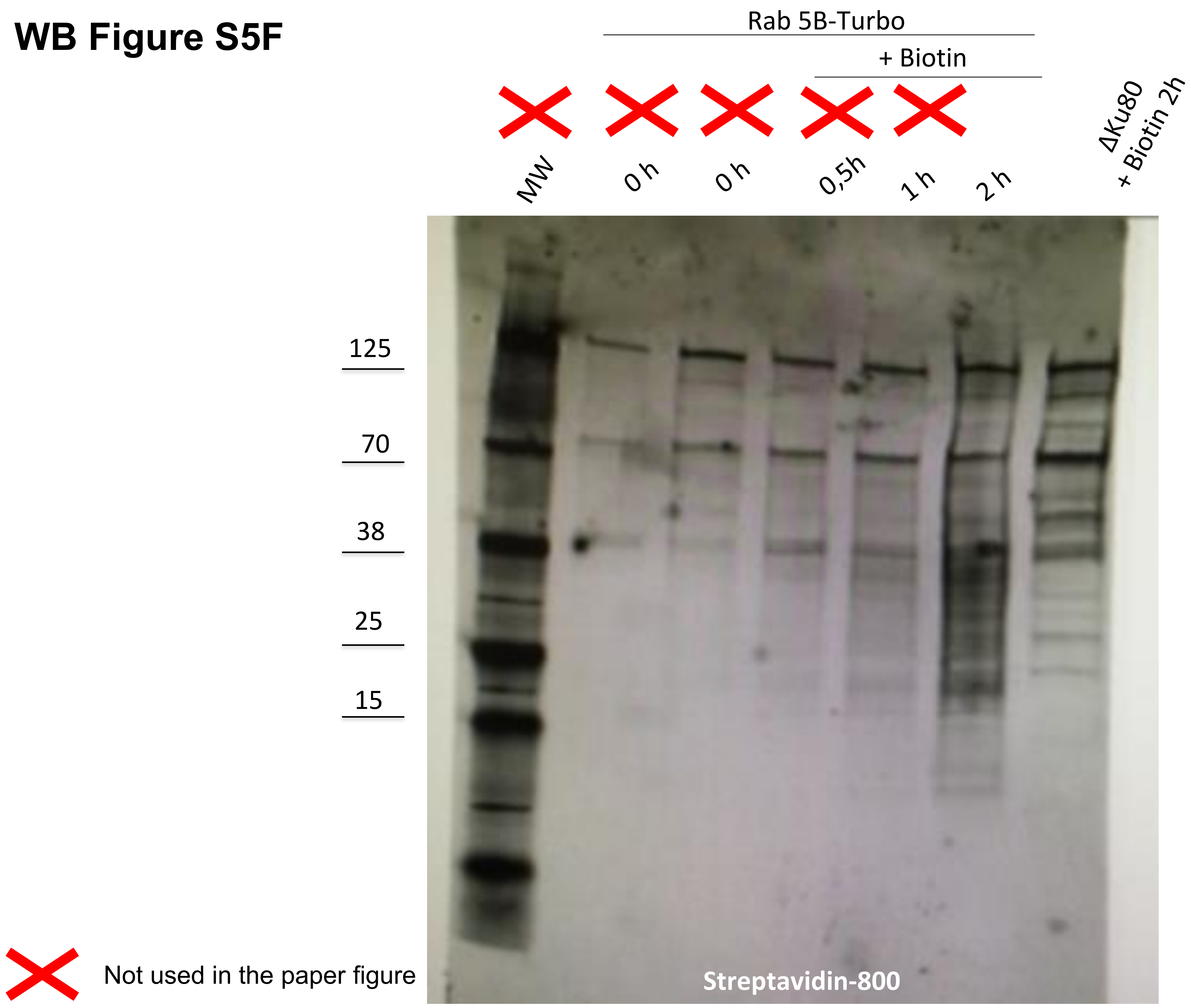

WB Figure S5G

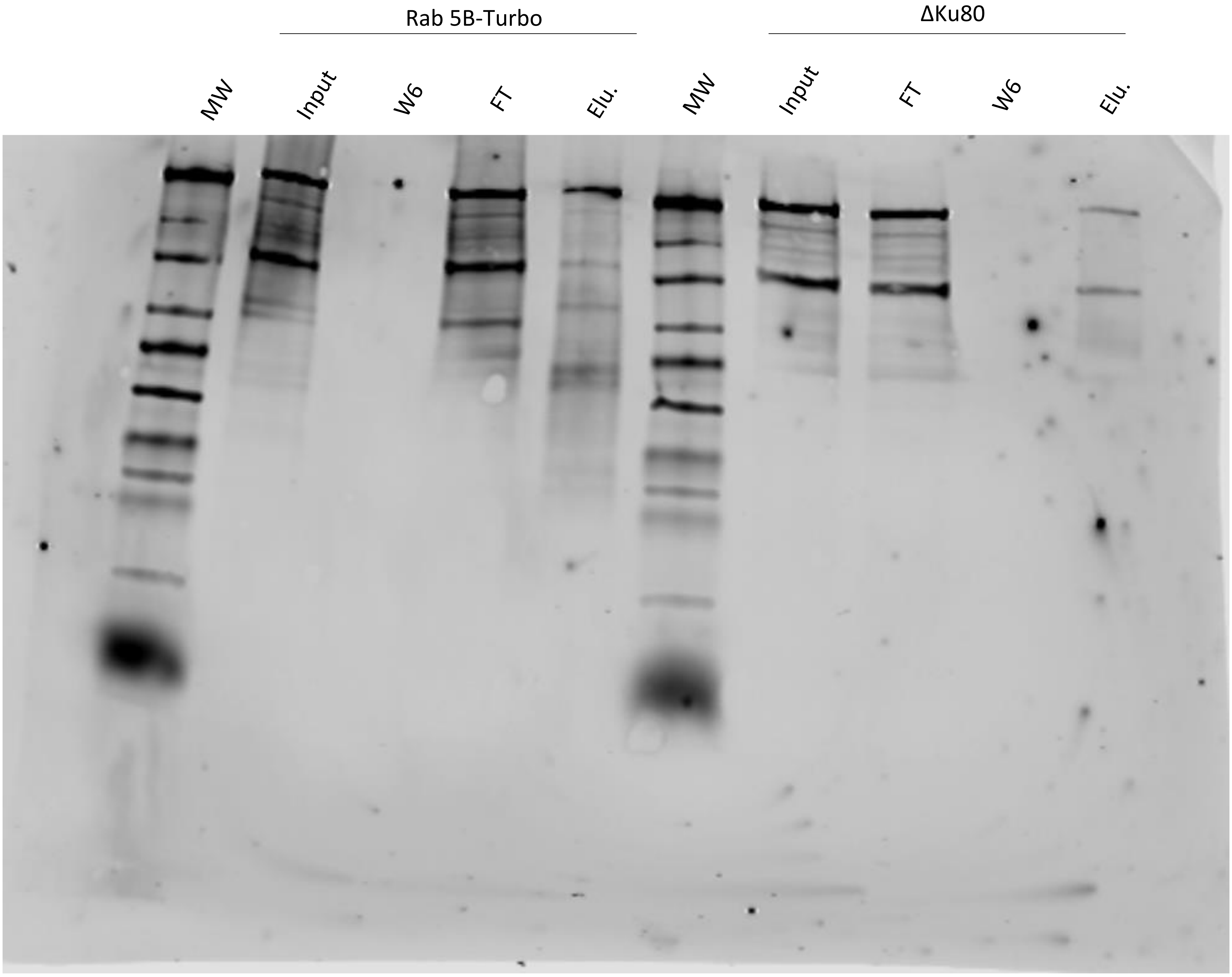

Supplement: S1 Raw Images — (PDF) [file pbio.3003415.s013.pdf]
