# Supplementary material for: Plasma membrane recycling drives reservoir formation during Toxoplasma gondii intracellular replication
Source: PLoS Biol. 2025 Sep 30;23(9):e3003415. doi: 10.1371/journal.pbio.3003415 (PMC12503318; doi:10.1371/journal.pbio.3003415)
Supplement: Attachment — Submitted filename: Response to Reviewers.docx [file pbio.3003415.s016.docx]

**Response to Reviewers**

We would like to thank the reviewers for their time, effort, and valuable comments, which have significantly contributed to improving the quality of our study. The revision has led to major changes in the article, notably the addition of the characterization of Rab5b-KD on the Toxoplasma lytic cycle requested by the editor. Two main and one new supplementary figures have been generated. A new section of the text is dedicated to the Rab5b analysis, and the discussion has been adapted accordingly to incorporate the new results.

Considering these changes, we propose to update the article’s previous title, “Toxoplasma gondii micropore is required for regulating the membrane reservoir during parasite replication”, to “Cycles of growth: plasma membrane recycling and reservoir formation during Toxoplasma gondii replication”, as we believe this better encompasses the data presented in the revised manuscript.

In addition, we have addressed the reviewers’ remarks; please find below our point-by-point responses.

Reviewer #1:

The intracellular protozoan Toxoplasma gondii divides by endodyogeny wherein two daughter cells form within a mother cell. The daughter cells develop their own specialized subpellicular membranes (the inner membrane complex) but utilize the mother cell membrane as the bud from the mother cell. Also, it has been previously established that daughter cells remain connected via a residual body and can share cytosolic contents. These peculiarities open several interesting questions about membrane sources and dynamics during parasite replication. By using thorough and quantitative analyses, the current study discovers that the plasma membrane is shared among parasites within the same replicative compartment (the parasitophorous vacuole), that at each cell division the PM is a hybrid of inherited material, recycled material, and de novo material, and that recycling of the PM is associated with a Rab5b positive compartment. Also, although (as somewhat expected) myoF is necessary for
the dynamic movement of endocytic vesicles it is not necessary for endocytosis. The authors also identify a so-called plasma membrane reservoir (PMR) that emanates from the PM and transiently precedes division at each cell cycle, and they suggest this material is reabsorbed in a micropore dependent manner. Overall, this work significantly advancing our understanding of PM dynamics in a system that has unique features compared to model organisms and exposes new biology that is relevant to other protozoa.

Main comments
This reviewer is uncertain of what the authors are suggesting with respect to the fate of PMRs. One the one hand, in the section beginning at line 293, they seem to indicate the PMRs are utilized as a source of membrane to facilitate expansion of the mother cell PM, thereby ensuring sufficient membrane for the budding daughters. This interpretation appears to be supported by video S2. One the other hand, in the section beginning at line 330 they suggest that the PMRs are reabsorbed via the micropore. Although their data shows that impairing micropore function results in more PMR, this is probably because of a block in the normal PM recycling process rather than reabsorption of the PMR itself. In this regard it might be more of an accumulation of excess membrane under a non-natural situation rather than a reabsorption of PMR. Perhaps there needs to be a clarification of terms (PMR vs excess membrane cause be defective recycling) to avoid confusion.

We thank the reviewer for the comment. We agree that further clarification is needed, as the term reabsorption is not appropriate and may lead to confusion.

Our data indicate that the PMR is most likely generated to facilitate membrane expansion during daughter cell budding, as shown in Video S2. Additionally, we show that the PMR varies in both position and size, as illustrated in Figure S7C. Increases and decreases in PMR size can be observed independently of any significant change in the overall size of the tachyzoite (see figure below, between T0 and T8:30). Given that membrane dynamics are ongoing, we believe these variations reflect the balance between secretion and endocytosis, a process that is critical for many membrane-regulating mechanisms. Here is the figure S7C

In the absence of K13, where micropore collapse impairs endocytosis, this balance is disrupted. The inhibition of endocytosis shifts the system toward an accumulation of secreted material. We believe this excess material contributes not only to an increase in the PM but also interferes with PMR formation. To support this hypothesis, we performed additional analyses and show that, upon K13 depletion, not only does the percentage of parasites with visible PMRs increase, but also the number of PMRs per parasite, their average size, and their total surface area (Figure 6E, F, G). We propose that disruption of the exo-endocytic balance alters PMR dynamics and ultimately contributes to the plasma membrane homeostasis defects we described in our previous study.

Accordingly, we have revised the manuscript. The previous section titled “The micropore controls the reabsorption of the membrane reservoir” has been modified and is now entitled “The balance between endocytosis and exocytosis is critical for PMR regulation” to better reflect these findings.

Minor comments
Figure 3d. some of the column labels are cut off.

We thank the reviewer for the comment. We assumed the reviewer was referring to the column in Figure 3C and have corrected it accordingly. We also updated Figure 4J, which contained the same error.

------------------------------------

Reviewer #2:

Here, the authors examine the dynamic and recycling of the plasma membrane in Toxoplasma gondii. Using FRAP experiments and SAG1-Halo as a proxy for PM dynamics, they nicely showed that the PM of T. gondii has a high fluidity within a parasite, and being also fluid between individual parasites of the same vacuole, which is easily explained by the well-described connection of PM of parasites within a vacuole. They set up a variety of different staining schemes and live-cell imaging to quantify and follow the different pools PM during replication. They observed a very dynamic system, including inheritance of the PM, and both endocytosis and exocytic pathways. Analysis of the potential players of the endocytic pathway, showed partial colocalisation with Rab5B, and a reduction of the traffic of endo-vesicles in absence of MyoF, without any disturbance of PM inheritance or homeostasis. They also described membrane loops, referred as plasma membrane reservoir PMR. This structure
is formed transiently, before the start of division, is reabsorbed, translocated to the posterior end of the parasite, where it becomes indistinguishable from the residual body. This structure is proposed to anticipate the future expansion of the PM required during the budding process of the daughter cells.

Major comments

1. The results and conclusions are sometimes difficult to follow. What is lacking in text and legends is a more detailed description of the experimental steps to help the reader evaluating the authors interpretation of the phenomena. For example, how long after labelling did they analyse parasites? or in figure 3A, what means « begining of intracellular development? » : is it staining of intracellular parasites or a pulse before invasion (figure 6)?

We thank the reviewer for this comment; we went through the text and the legends to add more details about the experiment to make it easier to understand.

The text was modified as follow: To test this hypothesis, we performed a modified triple labelling strategy, allowing us to independently label these sub-populations (Fig.3A). Freshly egressed parasites were first labelled for 1 hour with a membrane non-permeable dye (Halo Alexa-488), resulting in labelling of PM-SAG1. After washes, labelling with a membrane permeable dye was performed to labelled Int-SAG1 for 1 hours (Halo-Jan 646). After another round of washes, parasites were allowed to invade and replicate for 24h. To visualise n-SAG1, intracellular parasites were labelled for 1h with a new membrane permeable dye (Halo-Jan 549) (Figure 3B). Single stage parasites were selected to visualise n-SAG1 while limiting the amount which would have complete a cycle of exo-endocytosis.”

2. Several findings are confirmative. It has been already shown by several EM studies that the PM is shared between intracellular parasites within the same vacuole. We thank the reviewer for the comment. We are aware that some panels of the different figures are redundant with the actual literature like figure 1G or the indication of the endocytic vesicles in 2B. We chose to still put them in main figures instead of supplementary as we thought this would make it easier for the reader to follow. We have simplify the Figure 1 and Figure 2 to avoid too much redundancy with the literature.

What is really new is the analysis of the fluidity of the PM by FRAP experiments.

It is also known that the PM follows a cycle of endo- and exocytosis (Gras et al. PloS Biol 2019), and that depletion of K13 reduces the endocytosed vesicles (Koreny et al, Nat Comm 2023).

While our previous publication (Gras et al., PLoS Biol 2019) demonstrated that the plasma membrane undergoes cycles of endo- and exocytosis, this was shown only for extracellular parasites, relying on antibodies and general lipid dye. In the present study, we extend these findings to intracellular parasites, without permeabilization or relying on those antibodies and further demonstrate that the daughter cells inherit the plasma membrane of the mother cell.

Similarly, we are not repeating the characterization of K13's impact on endocytosis (Koreny et al., Nat Commun 2023), but instead analysing how disruption of the endo-exocytic balance affects PMR formation (Figure 6).

3. A direct link of the role of Rab5b with the cycle of endo- and exocytosis of PM has not been shown ; they showed only partial colocalization between SAG1 vesicles and Rab5b. The discussion on the potential role of Rab5b is very long, although there is little evidence to support rab5b's role.  Are the dynamics of the MP vesicles changed in the rab5b mutant?

We thank the reviewer for this comment, which raises a very interesting question.

We have now generated a Rab5b knockdown strain using the mAID system and characterized the effects of Rab5b depletion on the *Toxoplasma* lytic cycle, endocytosis, and vesicle trafficking. These results are presented in a new figure, and we hope that the additional data on Rab5b now provide a stronger foundation for the discussion.

4. The exact function of the PMR is not demonstrated, neither that the defect in its reabsorption leads ultimately to the death of the K13 mutant (as mentionned in the abstract). The death of parasites (previously demonstrated in absence of K13) might be due to a defect in endocytosis of key metabolites, as shown recently by Wan et al. Nature Comm 2023. The study is descriptive, the function of PMR remains speculative. If the PMR and its reabsorption serves as a reservoir for PM of daughter cells, why is the PM well visible and looks quite normal in the daugher cells of the K13 mutants ? other potential functions of the PMR might be proposed : a) to supply membrane at the posterior end of parasites for the connection of the daughter cells, which would explain why the K13 mutants are not connected, as shown in Koreny et al. Nat Comm 2023 ; and/or b) a function in establishing the tubulovesicular network that is initiated at the posterior end of the parasite ?

We thank the reviewer for this insightful comment and fully agree that the original text was too speculative regarding parasite death in the K13 knockdown (KD). We have revised the text to better align with the data and limit speculation.

Our intention in using the K13 KD was to investigate how altering the endo-exocytic balance impacts PMR formation. As noted in our response to Reviewer 1, the PMR is a highly dynamic structure that fluctuates in size and position. Like any membrane-associated process, its regulation likely depends on a balance between endocytosis and exocytosis. Disruption of endocytosis in the K13 KD disturbs this balance, favoring secretion, which results in the formation of more numerous and larger PMRs. Ultimately, the accumulation of these abnormal PMRs leads to the exacerbated plasma membrane (PM) alteration phenotype observed in K13-depleted parasites. We acknowledge that the term reabsorption was misleading and have reworded the text accordingly.

To address the reviewer’s question—why the PM appears well defined and relatively normal in the daughter cells of the K13 KD parasites—we clarify that the issue is not a lack of PMR, but rather an excess. The overabundance of PMR material leads to its accumulation across the PM, providing more than enough membrane for daughter cell budding. However, this surplus seems to interfere with proper membrane remodeling, disrupting the typical clean organization of the residual body. In Koreny et al., Nat Commun 2023, we similarly observed disorganized vacuoles where the residual body was no longer clearly identifiable; instead, cytoplasmic material was scattered between parasites, though they remained membrane-connected.

We also agree with the reviewer that the PMR may serve additional PM-related functions, such as supplying membrane to the posterior end to aid in daughter cell connection. This point has now been incorporated into the discussion, which reads as follows: “Taking our results into consideration, we hypothesize that PMR formation could be: (a) a process that anticipates the future expansion of the plasma membrane required during daughter cell budding; (b) an excess of membrane to support the intense endocytic activity occurring during replication; (c) a membrane reserve that facilitates the future connection of daughter cells after their emergence; or (d) a combination of these hypotheses.”

In contrast, with respect to the formation of the tubulovesicular network (TVN) or intravacuolar network (IVN), the complete absence of SAG1 signal in the parasitophorous vacuole (PV) suggests that the PMR is unlikely to contribute to this process. Proteins known to be involved in IVN biogenesis—such as the dense granule protein GRA2—exhibit a clear PV localization, unlike our SAG1 labelling.

Other comments

5. For figure 1 (FRAP experiment), the dye used is not indicated in legend and M&M. It is supposed to be a non-permeable one otherwise they would not be able to discreminate between diffusion at PM from neogenesis.

Yes, the dye used was a membrane non-permeable dye, Alexa 488. The M&M and the legend has been updated to provide this information.

6. For the measure of neo-SAG1, it is important to control that there is saturation of M-SAG1 with the first dye. In sort, that there is no binding of either external or internal SAG1 pools by subsequent 2nd dye application before replication, such that secondary labelling exclusively labelled the Neo-SAG1 pool. Same apply for the measure of Int-SAG1.

We thank the reviewer for this comment which raises a critical point of the experiment. The specificity of binding and saturation of the Halo tag is indeed crucial, and this has been demonstrated and published in Koreny et al., Nat Commun 2023.

Figure S2 (From Koreny et al. 2023):

Panel A illustrates two different labelling strategies: (I) Just one membrane permeable dye Oregon green (II) An additional membrane permeable dye TMR is used before the addition of the Oregon green. B and C are the image and quantification of the fluorescence signal in both cases. It is noticeable that the preliminary incubation with a first membrane permeable dye prevents the binding of a second and that the saturation by this first dye is complete.

Proper saturation under our experimental conditions is also demonstrated in the same study, where parasites incubated at 4°C show a clear separation between PM-SAG1 and Int-SAG1. This is observed when parasites are first labeled with a membrane-impermeable dye (PM-SAG1) followed by a membrane-permeable dye (Int-SAG1). For clarity, we have included a high-resolution image from Koreny et al., Nat Commun 2023, with updated labeling to match the nomenclature used in our manuscript.

PM-SAG1

Int-SAG1

Merge

DIC

4˚C

Labeling typically occurs within 10 minutes; however, we incubate our parasites for a full hour to ensure complete labeling and saturation of the targeted SAG1-Halo population.

7. Figure 2D, and conclusion lines 186-188. 'we found that loss of intensity after each round of replication is not as severe, when compared to PM-SAG1 (Fig.2E), suggesting that the secreted Int-SAG1 is inserted inside the PM. ' This conclusion is not intuitive and needs to be better explained.

The text has been modified to obtain better clarity. This now read as follow:

“The FI Int-SAG1 in the vesicles was decreased by ~twofold at each replication, passing from 100% to 58±19% between stages 1 and 2, then 32±5% at stage 4 and 18±7% at stage 8 (Figure 2G, green). This decrease in FI inside the Int-SAG1 vesicles suggests that less material is present within the vesicles. Two hypotheses could explain this reduction: (a) the material inside the Int-SAG1 vesicles is degraded, or (b) the material inside the Int-SAG1 vesicles is secreted.

To test these hypotheses, we measured the FI of Int-SAG1 located at the PM (Figure 2G, white). We found that, similar to PM-SAG1 and M-SAG1, the intensity decreased after each round of replication (Figure 2E). However, when comparing the signal evolution between PM-SAG1 and Int-SAG1 at the PM, we observed that Int-SAG1 FI decreased more slowly (Figure S3D). This slower decrease indicate insertion of labelled Int-SAG1 into the PM. As the dye used to labelled Int-SAG1 was removed and the excess washed off prior transfer to the parasite to host cells, the origin of signal must come from the intracellular vesicular stock, supporting hypothesis (b)—that Int-SAG1 vesicles are secreted to the PM during replication”

Comparison between the evolution of the membrane signal between the PM and INT SAG1 have been added in supplementary figure S3D to facilitate the comparison and understanding.

8. The demonstration that parasites share PM continuity within a vacuole is not new. What is really new is the analysis of the fluidity of the PM by FRAP experiments. Change the title of the secetion Line 74 : 'Parasites within a parasitophorous vacuole share a continuous plasma membrane ' to reflect better the novelty of the conclusions of this section.
9. Lines 118-119 : Electron microscopy does not allow to examine if the PM is exchanged between two parasites, it just shows continuity.
10. Figures 1H-J. and corresponding text Lines 121-126 : For non expert in the Toxoplasma field, it is necessary here to introduce what is the residual body, and the presence of F-actin at this location, and what is the Cb-SNAP. Panel H : the enlarged merge does not correspond to the individual enlarged SAG1-HALO, Cb-SNAP or GAP45, or the magnification is not the same for all chanels. The PM covering the residual body is not obvious in the IFA, while it was already well shown by EM, and confirmed here in Fig. 1G.
We thank the reviewer for these comments. They have been regrouped as they are all concerning the same section of the text and the article. An important rework of the text has been performed to match all the reviewer comments. The title of the section has been changed, more details have been put about the F-actin, chromobies, residual body and SNAP, the figure was updated and part transfered to supplementary to make the Figure 1 more fluid to read and less redundant with the literature. This whole first section now read as follow:

“During replication, it has been demonstrated that daughter cells remain connected via an intravacuolar network, which is essential for the exchange of material and signals between individual parasites (24). The central point of this connection is referred to as the residual body (25). This residual body, along with the connection between parasites, is supported by F-actin filaments, which serve multiple functions, ranging from parasite synchronization to organelle trafficking. These F-actin filaments were visualized using camelid nanobodies called Chromobody (Cb), expressed inside the parasite (26).

FRAP experiments on vacuoles containing multiple parasites (expressing a cytosolic GFP), showed a fast recovery after bleaching and therefore efficient exchange of material from one parasite within the PV to another (26, 27). This sharing of cytoplasmic content is lost in mutant parasites where the intravacuolar network is absent, for example in a conditional null mutant for act1 which cannot form the F-actin filaments (28).

As previously demonstrated, parasites within a single PV are connected at the posterior end, with a continuous PM and a shared cytoplasm (Figure 1G). The PM also covers the newly formed residual body where F-actin filaments are located (Figure 1G, S2). This coverage of the residual body is maintained throughout replication, as demonstrated by the colocalization of SAG1 and F-actin filaments. The F-actin filaments were imaged using an actin-chromobody fused to a SNAP tag (Cb-SNAP). The SNAP tag technology, similar to the Halo tag, allows visualization in different colours using specific dyes. We observed in 93±6% of the vacuoles, even at later replication stages (Figure S2B, C). This maintained connection should allow the transfer of proteins between parasites within the same vacuole.

To assess the potential exchange at the PM between parasites of the same vacuole, FRAP experiments were performed on large vacuoles, where half of the parasites were bleached (Figure 1H). We observed recovery of the SAG1 signal at the surface within 5-10 minutes. These results illustrate that the membrane is fluid between individual parasites of the PV. The longer recovery time, when compared to the experiments on individual parasites can be explained by the intravacuolar network, potentially representing a bottleneck for diffusion. Nevertheless, this experiment demonstrates that parasites within a PV share the same PM content. »

11. Line 135 : precise inheritance of the PM in the title 'Inheritance of the maternal material during replication'
The text has been edited as suggested.

12. One can wonder in Fig. 3a, how the authors where able to count the number of vesicles in the residual body, since the labelling appears like a messy staining ?

We thank the reviewer for this comment. We agree that the picture with their current exposition is not helping to visualise the residual body. The figure has been updated to allow a better appreciation on how it is possible to observe the vesicles inside the residual body, with a zoom in on the residual body.

13. On figure 2C, we see an accumulation of neo-synthetized vesicles in vacuoles containing 8 parasites, while none are visible in the vacuole containing one or two parasites. What does it mean?

Here, two factors are to be taken into consideration to explain this visibility of the vesicles in the large stage vacuole.

First and most important factor, the intensity of all the pictures of n-SAG1 are fixed at the same FI level between stage 1 to stage 8 to allow a good comparison. It was set using stage 2 parasite FI. While it allows a good visualisation of the increase of n-SAG1 present during replication, in counterpart it led to a low exposure to stage 1 parasite and an over-exposition of stage 4 and 8. The increase of the vesicles visible is in our opinion in part due to this difference in exposition. If Stage 1 vacuole are also over-exposed, vesicles can also be observed. They are not visible overwise due to the difference in amount between the SAG1 at the PM and in those vesicles.

Second, the n- SAG1 will enter the cycle of secretion and recycling starting the moment when it is produced at stage 1. We also believe that the amount of n-SAG1 in recycling vesicles increase while replication continue as more and more n-SAG1 will have time to undergo secretion and endocytosis.

We hope that these explanations will clarify the situation.

14. Line 194 : mix of Endo-SAG1 AND de novo-SAG1.

The text has been edited as suggested.

15. Line 211 : A total of 225 Endo-SAG1, 722 Int-SAG1 and 337 n-SAG1 VESICLES were observed.

The text has been edited as suggested.

16. Lines 234-236 : Ref 28 and 29 do not correspond with text.

We thank the reviewer for this comment, this error has been corrected.

17. in 7b legend line 940 '… with Rab5b appearing to play a role in this process'. The role of Rab5b has not been demonstrated.

We thank the reviewer for this comment, a whole now figure on the function of rab5b have been added.

18. in 7a, the successive fluorescence images of a duplication cycle show a PMR in all cases. This is contradictory with the result in fig5 E where the percentage of vacuoles showing a PMR is about 10 %…

We thank the reviewer for this comment. This figure represents the evolution of a parasite during its first replication cycle. PMR are observed in about 95% of the parasite undergoing replication.

Yet, the comment about the 10% of vacuole with visible PMR highlighted that better explanation is needed. To increase the clarity of Figure 5, new quantifications were performed and the text update.

We now insist more on the importance to distinguish between unsynchronised and synchronised parasite population. As we used both pulse invasion (synchronised) for live replication and non-plus invasion (unsynchronised) for obtaining the different stages of parasite for the quantification of the signal intensity during our assay and we observed that number of simultaneous PMR visible varied depending on the technic used. This is due to the transient nature of the PMR and we thought it was important to highlight it for person interested to replicate the assay.

The snapshot of unsynchronised parasites after 24 hours of replication in host cells shows that approximately 10% of vacuoles exhibit a PMR. While this percentage appears relatively low, it is crucial to consider that parasites do not invade or replicate simultaneously or at the same rate, leading to variations in vacuole developmental stages.

Even with live microscopy with pulse invasion, where parasites are allowed to invade for just one hour, the parasite are not perfectly synchronised. This is visible in classical replication assay where after performing such pulse the distribution of parasite stages varies between stage 1 to 16 with a maximum around stage 4. During the 12h recording within the different fields of view, the amount of visible PMR at each time frame varied from 0 to 80% with an average maximum of about 40% of the tachyzoites forming a PMR at the same time, which is coherent with the fact that all the tachyzoites are not replicating at the same time too.

Using expansion microscopy on RH-parasites, up to 80% of the parasite were observed to form a PMR simultaneously in a synchronous population. We think that this difference with is related to the expansion of nascent /smaller PMR which cannot be observed with the resolution of the widefield microscope used for live imaging.

On the other hand, virtually all the tachyzoites that undergo replication were observed to form a PMR.

The figure 7a illustrate this formation of PMR in parallel to the formation of the daughter cells and their budding of a single replicating tachyzoites.
